# Personalized, digitally designed 3D-Printed protein substitutes: Advancing medical food for PKU patients

**DOI:** 10.1016/j.crfs.2026.101314

**Published:** 2026-01-19

**Authors:** Fidaleo Marcello, Zohreh Baratian Ghorghi, Giovanni Luca Russo, Annachiara Ferraioli, Silvana Cavella, Rossella Di Monaco

**Affiliations:** a Department of Agricultural Sciences, Unit of Food Science and Technology, University of Naples Federico II, Portici, 80055, Italy; b Department for Innovation in Biological, Agro-Food, and Forest Systems, University of Tuscia, Viterbo, 01100, Italy; c Department of Agricultural, Forest and Food Sciences, University of Turin, Turin, Italy

**Keywords:** 3D printing, Glycomacropeptide, PKU protein substitute, Rheology, Sensory evaluation

## Abstract

Phenylketonuria (PKU) is a rare inherited metabolic disorder requiring lifelong restriction of phenylalanine (Phe) intake. This study aimed to develop and characterize 3D-printed protein substitute medical foods based on glycomacropeptide (GMP), a natural Phe-free peptide derived from cheese whey, tailored for PKU patients. Cocoa butter was used to facilitate extrusion performance. Three inks containing cocoa butter, tagatose and pineapple powder, with varying concentrations of GMP and selected amino acids, were developed to yield protein-equivalent contents ranging from 24 % to 31 % (w/w) and Phe contents respectively between 1.76 and 1.79 mg per gram of protein equivalent; these formulations were evaluated for printability, thermal and rheological properties, mechanical strength, wetting behavior, color and sensory attributes. All food inks achieved high printing accuracy and stable structures. Tempering imparted structural integrity and favorable physicochemical properties to the inks; this stability was maintained post-3D-printing, as evidenced by wetting behavior analysis. Thermal and rheological analyses demonstrated that higher GMP concentrations enhanced the viscosity and thermal stability of the inks, while mechanical testing of the 3D-printed structures indicated improved matrix rigidity with increased GMP content. Sensory evaluation of the obtained snacks using Temporal Dominance of Sensations (TDS) showed distinct oral perception profiles across formulations, with higher GMP content linked to increased adhesiveness and prolonged flavor perception. However, inks with higher tagatose levels exhibited more prominent sweetness. The findings suggest that 3D printing may be a feasible approach for producing customized, palatable medical foods for PKU patients, with GMP serving as a key functional protein.

## Highlights

• 3D-printed GMP-based protein substitutes tailored for PKU management.
• All inks achieved high print accuracy; structures remained stable post-printing.
• Higher GMP increased viscosity, rigidity, and thermal stability of inks.
• GMP enrichment prolonged adhesiveness and flavor.

## Graphical abstract

**Figure:**
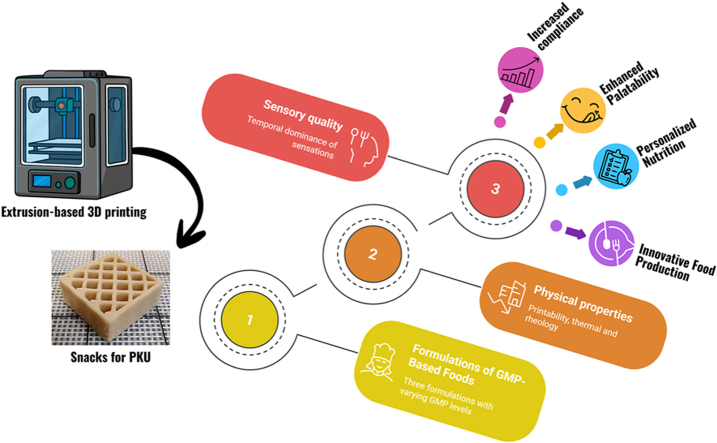

## 1 Introduction

Phenylketonuria (PKU) is an inherited metabolic disorder caused by a deficiency of phenylalanine hydroxylase (PAH) which normally converts phenylalanine (Phe) to tyrosine. Deficiency of this enzyme leads to an increased production of phenylketone bodies (hence phenylketonuria) and accumulation of Phe in the blood and brain, causing severe and irreversible neurocognitive impairment (MacDonald et al., 2020). The prevalence of PKU varies widely among countries, but it is 1:10,000 in Europe, with a higher rate in some countries including Italy (Burlina et al., 2021). The number of individuals with treated PKU is estimated at 50K worldwide (Soltanizadeh and Mirmoghtadaie, 2014).

PKU is successfully managed by a protein-free diet supplemented with Phe-free L-amino acid (AA) supplements. The prescribed PKU diets typically include only restricted amounts of vegetables and fruits, special food with low protein content and an enriched formula to provide other essential AAs (typically added with vitamins and minerals or also with fat and carbohydrates) (Soltanizadeh and Mirmoghtadaie, 2014).

Traditional medical foods are mainly based on mixtures of free amino acids. Although nutritionally effective, these products are frequently described by patients and caregivers as having an unpleasant taste and odour, low palatability, and limited variety of formats (Proserpio et al., 2019). The need for multiple daily doses, the low satiating power of free amino acids and the social visibility of these products further contribute to poor adherence, especially in adolescents and adults, and to difficulties in maintaining good long-term metabolic control. Improving the sensory quality, convenience and personalization of protein substitutes is therefore a key clinical need in PKU management (Proserpio et al., 2019).

Casein glycomacropeptide (GMP) is a 64-amino acid peptide from cheese whey which is rich in specific essential amino acids and is the only known natural Phe-free protein source (Lim et al., 2007; Van Calcar and Ney, 2012). This product has been demonstrated to be efficient in the management of PKU patients and there are several medical foods based on GMP available commercially, like PKU Sphere™ (Nestlé Health Science S.A., Switzerland).

Recent studies carried out in Italy (Proserpio et al., 2018, 2019) involved Italian subjects affected by PKU, evaluating the acceptability of differently flavored formulas made up of either GMP or amino-acid mixtures. Results demonstrated a higher acceptability of GMP samples, as characterised by sweet and mild taste, mild odour, and natural colour, compared with amino acid formulations. The authors highlighted that the above-mentioned sensory attributes should be considered as key factors influencing subjects’ satisfaction. GMP has such functional properties as solubility in acids, forming foams or gels, and proper heat-stability that make it suitable for use in semisolid foods such as puddings and beverages (Lim et al., 2007). In chocolate beverages produced with GMP, this peptide also enhances the chocolate flavour helping to camouflage the dairy flavour of GMP (Van Calcar and Ney, 2012).

In the context of personalized nutrition, the terms “customized food formula” have been introduced (Severini and Derossi, 2016) to refer to the preparation (at home) or the production (at industrial level) of new food formulations having nutrients and functional compounds necessary to prevent diseases or to reduce the risk for each subject (or subjects’ category) who exhibit a susceptibility to diseases. The medical counterpart of personalized nutrition is personalized medicine, broadly defined as “providing the right treatment to the right patient, at the right dose at the right time” (Beer et al., 2021).

Three-dimensional (3D) printing is an interesting technology able to produce materials with desired shape, dimension, and structure properties, thus it has been a topic of interest to both commercial and academic researchers in the past decade (Voon et al., 2019). A considerable volume of research has been published focusing on developing food inks for extrusion-based printing beyond conventional materials such as chocolate, resulting in a wide range of printable and edible materials. Five categories of printable materials particularly have been identified: confectionery inks such as chocolate or dough, dairy inks based on milk or its derivatives, edible hydrogel inks, as well as inks created from plants or meat. These materials have been grouped into two broader categories: natively extrudable (i.e. confectionery, dairy, hydrogels) versus non-natively extrudable (i.e. plants, meat).

In the above context, the application of 3D printing allows us to build personalized foods by depositing nutrients and functional compounds, or soft materials obtained by their mixture (Severini and Derossi, 2016). Challenges and scenarios of 3D printing as a promising tool for personalized medicine have already been investigated (Beer et al., 2021). Examples include the production of pediatrics orodispersible printlets of hydrochlorothiazide (Eduardo et al., 2021), pediatric-friendly chocolate-based dosage forms for the oral administration of both hydrophilic and lipophilic drugs (Karavasili et al., 2020), or medicinal gummies (Herrada-Manchón et al., 2020; Tagami et al., 2021). These works showed that 3D printing in the pharmaceutical field allows the preparation of personalized medicine that could solve a variety of pharmaceutical formulation challenges for pediatric population such as dose flexibility, patient compliance and taste masking (Januskaite et al., 2020; Goyanes et al., 2019; Rodríguez-Pombo et al., 2024; Carou-Senra et al., 2024).

Based on these considerations, the use of 3D printing technology to produce personalized enriched medical food for the supplementation of AAs in patients affected by PKU appears very promising. In particular, it could contribute to solving compliance problems by making food with tailored and improved sensory properties and accurate nutritional content available to PKU patients in both home and hospital settings. This study aimed to develop and characterize a 3D-printed food prototype specifically designed for individuals with PKU as a protein substitute. In particular, the study focused on evaluating the printability, thermal and rheological properties, mechanical strength, wetting behavior, color and sensory attributes of different food inks to assess their suitability for 3D printing and their acceptability as palatable, medical foods for PKU patients.

## 2 Materials and methods

### 2.1 Materials

XPhe enjoy^20^ GMP (neutral flavor), a powdered protein supplement with a reduced Phe content containing 60 % of protein equivalent, was supplied by Mamoxi s.r.l. (Torino, Italy). It is based on GMP, with the addition of highly purified L-amino acids Alanine, Arginine, Aspartic acid, Glycine, Histidine, Leucine, Lysine, Tryptophan as well as Tyrosine and enriched with soluble fibres from partially hydrolysed corn starch (polydextrose). Tagatose, a low-calorie sweetener and bulking agent classified as GRAS by the Food and Drug Administration for use in food and beverages, was kindly donated by Bonumose Co. (Charlottesville, VA, USA). Pineapple juice powder enriched with 60 % w/w maltodextrin, sunflower lecithin and cocoa butter (60 % saturated fatty acids, melting point 33–36 °C) were kindly donated by OPTIMA S.p.A. (San Clemente, RN, Italy).

### 2.2 Ink preparation

Three food inks were developed by using the following ingredients: cocoa butter, XPhe enjoy^20^ GMP (neutral flavor), pineapple juice powder, tagatose, and sunflower lecithin. Table 1 shows the recipes for the three inks. Ink ingredients were refined in a Premier chocolate refiner (model no. 507, Diamond Custom Machines Corp., Hillsborough, NJ, USA) (capacity: 3.5 kg/batch) for 5 h. Lecithin was dissolved in the cocoa butter previously melted at 45 °C and the resulting mixture was poured into the refiner. Then, the machine was turned on and the solid ingredients were added gradually to avoid excessive viscosity of the paste. To this aim a heat gun was used to apply heat to the external surface of the refiner as needed. The temperature of the refining mixture was about 50 °C after 45 min from the operation's beginning due to the stones' friction and remained at about the same value throughout refining.

**Table 1:** Composition of the three food inks developed.

Ingredients	Formulation (wt %)		
	A	B	C
Cocoa butter	32	32	32
Pineapple juice powder	10.7	10.7	10.7
Tagatose	16.2	10.2	4.2
XPhe enjoy^20^ GMP	40	46	52
Lecithin	1.1	1.1	1.1

After the refining process, the mixture was molded into polycarbonate moulds and kept in the fridge for one night. The next day, after taking aside some product to be used as a non-tempered sample, the remaining product was tempered by hand operation as follows. First, the product was split into three parts of identical weight. Then, 1/3 of the product was used as the seeds and added in the solid state to the remaining 2/3 of it previously melted at 45–50 °C. The mixture was stirred by hand in a bowl until the temperature reached 27 °C. Finally, the mixture was reheated in a microwave oven to reach 29–30 °C, formed into the abovementioned moulds and into cylinder-shaped silicone moulds (diameter of 2.5 cm, length of 38 cm) (as ink to be printed by the 3D printer). The moulds containing the product were then placed in plastic bags, sealed, and kept overnight in a refrigerator (4 °C). The next day, the molded products were removed from the moulds and stored in sealed plastic bags at room temperature for further chemical-physical characterization and for 3D printing studies.

### 2.3 Macronutrients, energy and phenylalanine content

Macronutrient (fat, carbohydrates, protein, fiber), energy, and phenylalanine content of the formulated inks were calculated by aggregating the contributions of each ingredient weighted according to its proportion in the formulation. For each ingredient, nutrient and energy values were sourced from the manufacturer's technical sheets. The phenylalanine content was estimated for the pineapple powder from its protein content by using a phenylalanine/protein ratio of 27.4 mg of Phe/g of protein (Kim and Boutin, 2014; obtained as the average value of 27 entries concerning pineapple), for lecithin by considering its protein content by using a phenylalanine/protein ratio of 52.4 mg of Phe/g of protein (Kim and Boutin, 2014; obtained as the average value of 20 entries concerning sunflower seed) and for XPhe enjoy^20^ GMP from the product nutritional label (0.1 g of Phe/100 g of product). The following ingredients were considered free from proteins and phenylalanine: cocoa butter (listed as foods with zero Phe content by Kim and Boutin, 2014), tagatose. The energy content of D-tagatose was assumed as equal to 1.5 kcal/g (Ortiz et al., 2024). The above nutritional data were collected for some commercial protein substitutes, included in this study for comparative purposes. We considered commercially available ready-to-eat protein supplements in the form of snack bars for the dietary management of PKU: GOLIKE tropical bar (APR Applied Pharma Research SA, Arzo, Switzerland), Glyactin complete 15 Fruit frenzy (Ajinomoto Cambrooke, Northampton, MA, USA), GMP UP Afenil bars (PIAM Farmaceutici S.p.A., Genova, Italy), XPhe Hello (metaX Institut für Diätetik GmbH, Berlin, Germany) (Russo et al., 2025).

### 2.4 Thermal properties

The thermal transitions of the inks were characterized using a differential scanning calorimeter (DSC Q200, TA Instruments, Waters, LLC, USA). Each sample, weighing 8–12 mg, was placed in an aluminum pan and hermetically sealed. A hermetically sealed empty aluminum pan was used as the reference. The ink sample was maintained at 0 °C for 1 min, then heated at a rate of 10 °C/min up to 50 °C and held at this temperature for 1 min. Subsequently, the sample was cooled at a rate of 10 °C/min down to −40 °C and maintained at this temperature for 20 min, followed by heating at 10 °C/min up to 20 °C, where it was held for 5 min. Data were collected and analyzed using the TA Universal Analysis 2000 software (version 2.8.394), which provided measurements of heat flow (mW/g) as a function of temperature (T, °C). Key thermal parameters for first-order transitions were determined, including the onset temperature (T_onset_, °C), the peak temperature (T_pk_, °C), the end-set temperature (T_endset_, °C), and the specific enthalpy (ΔH, J/g), calculated as the area under the melting/crystallization peak divided by the sample weight. Five replicates were performed for each food ink.

### 2.5 Rheological properties

The rheological properties of the formulated inks were measured using a rotational stress-controlled rheometer (HAAKE MARS, Thermo Scientific, Karlsruhe, Germany) equipped with a parallel plate geometry (Ø = 60 mm, gap = 1 mm). Approximately 3 g of each melted ink was carefully placed on the lower plate of the rheometer, ensuring uniform coverage of the entire surface. The upper plate was then lowered gradually to a 1 mm gap, and any excess was removed with a spatula to achieve consistent sample loading. Prior to testing, each sample was maintained at 45 °C for 5 min to ensure thermal equilibrium. A temperature sweep test was conducted under static conditions at a constant shear rate of 1 s^−1^, while the temperature was decreased from 45 °C to 10 °C at a rate of 2 °C/min. The experimental results were recorded as viscosity (η, Pa∙s) versus temperature (T, °C). Each of the measurements was performed in triplicate to ensure reproducibility.

### 2.6 3D printing process

A Procusini® 5.0 3D food printer based on layer-on-layer food production technology, suitable for both hot and cold extrusion, was used for this project (Print4Taste GmbH, Germany). The printer could preheat the stainless-steel cartridge to the pre-set temperature in the range of 0–60 °C and for a specified amount of time before printing. Before starting to work with the 3D printer, the room temperature was lowered to 22–23 °C by using air conditioning. The ink in the form of a cylinder was placed in the cartridge of the machine, and the printing program was started. Pre-heating temperature and time were set respectively at 32 °C and 15 min. The distance between the printhead and the machine's mat screen (gap height) was adjusted manually by acting on an adjusting screw.

As a model object for 3D printing, a parallelepiped of dimensions 2.5x2.5 × 1.5 cm was chosen. The object was designed by using Fusion 360 CAD software (Autodesk, Inc. San Francisco, USA) and the corresponding drawing file was exported in STL format. The slicing phase and the creation of the G-code file were carried out using the online Procusini Club platform (Print4Taste GmbH, Germany). The infill rate was set to 40 %, the number of bottom, top, and wall layers was set to 0, 0 and 2 respectively, while the other printing parameters were set automatically by the platform as default values for the chosen ‘3D objects, Own stl files’ application.

### 2.7 Color measurements and printing accuracy

The printing accuracy and colorimetric properties of the printed cuboids were analyzed using a visual analyser (model VA400 IRIS, Alpha MOS, France) equipped with a charge-coupled device (CCD) camera featuring a resolution of 2592 × 1944 pixels and a 24-bit color depth. The camera, fitted with a Blaser Fujion 25 mm lens, was housed in a box with top and bottom illumination provided by 4 × 4 white LEDs. The lighting system was stabilized for 15 min prior to use to ensure consistent illumination. Images were acquired in raw format and processed in the RGB color space, then converted to the CIE L∗, a∗, and b∗ parameters using Alphasoft software version 16.0 (Alpha MOS, France).

The analyzed parameters included:

L∗: lightness, ranging from 0 (black) to 100 (white).

a∗: coordinate representing the transition from green (−) to red (+).

b∗: coordinate representing the transition from blue (−) to yellow (+).

The analysis was conducted in triplicate for each lateral side of the cuboids, with three cuboids analyzed per formulation. Samples were evaluated immediately after printing to avoid alterations due to storage conditions. The color difference between inks (ΔE) was calculated using Equation (1) applied to the mean colorimetric coordinates:

Eq. (1) $${\Delta E}_{ij}=\sqrt{{\left(L_{i}^{*}-L_{j}^{*}\right)}^{2}+{\left(a_{i}^{*}-a_{j}^{*}\right)}^{2}+{\left(b_{i}^{*}-b_{j}^{*}\right)}^{2}}$$

where L_*i*_∗,a_*i*_∗,b_*i*_∗ and L_*j*_∗,a_*j*_∗,b_*j*_∗ are the mean CIELAB coordinates of samples *i* and *j*, respectively. In this study, ΔE values were calculated pairwise between the three printed objects (A, B, and C).

The printing accuracy of the formulated inks was assessed through image analysis. Images of the printed objects were captured using the visual analyzer and subsequently processed with ImageJ software (Version 1.41) to evaluate the printed cross-section and the infill pattern. For printing accuracy, the actual printed area was measured and compared with the dimensions of the designed cuboids set during the printing process. The accuracy was determined using the method described by (Bareen et al., 2021), considering the dimensional variations in the printed objects. For infill accuracy, the area corresponding to empty spaces within the printed structure was quantified and compared with the predefined infill percentage of 40 %. All measurements were performed with five replicates at 25 °C to ensure reproducibility.

### 2.8 Contact angle measurement

Contact angle measurements were performed on tempered and non-tempered inks and on printed objects obtained from tempered inks to assess the ability to retain the structure imparted by the tempering operation after printing. Measurements were performed by the sessile drop method by using an automated optical tensiometer (model Theta flex, Biolin Scientific, Espoo, Finland). Basically, the instrument consisted of an automated pipettor, a sample holding plate, and a high-speed camera equipped with a microscope objective. 5-μL deionized water drops and 300 μL plastic pipettor tips (Neptune, Germany) were used for the measurements. Eight contact angle measurements were acquired for each sample, and the resulting values were averaged. All samples were kept in the measurement environment for at least 1 h before contact angle measurements to ensure they adapted to the room temperature conditions. Data were analyzed by using the instrument's proprietary software (OneAttension software, version 4.1.2).

### 2.9 Mechanical properties

The mechanical properties of the printed objects were assessed through compression tests conducted using an Instron Universal Testing Machine (model 4467, Instron Ltd., High Wycombe, UK). The printed objects used were cuboidal in shape, with a height of 1 cm, a cross-sectional area of 160 mm^2^, and an infill of 40 %. Tests were performed using a 60-mm diameter aluminum plate and a 1-kN load cell, selected based on the estimated maximum load during testing. Data acquisition was carried out using the Instron Series IX software (Instron Ltd., High Wycombe, UK).

The crosshead speed was set to 50 mm/min, and the maximum deformation to 50 %. Ten compression tests were performed for each formulation of the printed object.

From the stress-strain curves, two mechanical properties were estimated: breaking stress (σ_f_, Pa) and Young's modulus (E, MPa). Breaking stress was determined as the maximum load the sample could withstand before failure, while Young's modulus was calculated from the initial linear region of the stress-strain curve, using the following equation:

Eq. (2) $$E=\frac{\Delta \sigma }{\Delta \epsilon }$$

where Δ*σ* is the change in stress (Pa) and Δ*ϵ* is the corresponding change in strain, calculated as the ratio of the change in length to the initial length of the sample. The data were analyzed to evaluate the formulation effect on the stiffness and hardness of the printed cuboids.

### 2.10 Sensory evaluation

A panel of eight trained assessors (6 females, 2 males; mean age 28 years) evaluated the printed object in the Laboratory of Sensory Analysis at the Department of Agricultural Sciences (University of Naples). The assessor's selection was based on their ability to distinguish fundamental tastes, recognize odors and discriminate among different textures, ensuring consistent evaluations across the study (five selection sessions of 2 h each). Prior to the evaluation, the assessors underwent a specific training phase focused on recognizing and identifying the selected sensory attributes. In accordance with the Temporal Dominance of Sensations (TDS) methodology, training did not involve intensity scaling, as assessors were instructed to identify only the dominant attribute at any given time, without quantifying its intensity. The training phase (five training sessions of 1 h each) also ensured that assessors had a consistent understanding of the terminology and application of the TDS method to maintain methodological reliability (Galmarini et al., 2017). To develop a tailored sensory profile for the products under investigation, five focus group sessions were conducted. The aim of these sessions was to comprehensively characterize the sensory attributes of the food inks and to identify those that could discriminate between the different inks. Based on the focus group outcomes, six attributes emerged: sourness, adhesiveness, meltability, sweetness, hardness, and overall flavor. The TDS analysis was performed following established protocols (Di Monaco et al., 2014), aided by data acquisition software (Smart Sensory Solution, Italy). The TDS procedure captured dominant sensations over time among the attributes identified during the focus groups. During the TDS sessions, the assessors evaluated the sensory dominance of the selected attributes for each printed object over standardized time intervals (90 s). Each sample was evaluated in five independent replicates per assessor, ensuring sufficient data for statistical reliability. Sensory data were recorded as dominance rates (DR%) for each attribute, calculated as the percentage of times an attribute was cited as dominant by the assessors at each time point. The DR% data were plotted against standardized time (%), and data smoothing was applied to generate TDS curves. The resulting TDS curves included the two critical thresholds, chance level (*P*0) and significance level (*Ps*). Attributes with DR% exceeding the significance level were considered significantly dominant, while attributes with DR% between the chance and significance levels were interpreted as trends toward dominance (Galmarini et al., 2017).

### 2.11 Statistical evaluation

The data were analyzed using IBM© SPSS© Statistics software Ver. 23 (SPSS, Inc., Chicago, IL, USA). Analysis of variance (ANOVA) was performed to determine the statistical significance of the independent variable studied, followed by post-ANOVA multiple comparisons to compare the different levels of the independent variable (Fisher's method).

TDS curves were generated via the statistical analysis program XLSTAT Version 2019.2.2 (Boston, USA) through the sensory data analysis package. For every time interval and each attribute, pairwise comparisons between the three inks (A vs B, A vs C, and B vs C) were conducted using the McNemar test through Python (v.3.10) with the stats models library. The test was applied to binary dominance data, where a value of “1” indicated that the attribute was dominant at a given time point and “0” indicated otherwise.

All tests for statistical significance were run at a significant level α of 5 %.

## 3 Results

### 3.1 Macronutrients, energy, and phenylalanine content

Table 2 shows the nutritional content and Phe amount of the three snacks produced in this work and some other commercially available protein supplement snacks for PKU. The developed snacks present higher protein content in comparison to the commercial products (except the case of ink A vs. XPhe Hello). The amount of Phe of the snacks is also higher than commercial products, but when referred to the protein content, the ratio Phe/protein varies between 1.76 and 1.79 mg/g and is in line with the interval 1.26–2.11 mg/g estimated for the commercial snacks. Therefore, the higher absolute Phe content observed in the developed snacks reflects their higher protein content rather than a higher phenylalanine density, as confirmed by the comparable Phe/protein equivalent ratios. For the other macronutrients, the fat/carbohydrate ratio is higher for the developed snacks, while the sugar content is much lower. The energy content, mainly influenced by the fat content, is much higher for the developed snacks. Basically, the nutritional content reflects the fat-based nature of the developed products while the commercial ones are carbohydrate and sugar-based.

**Table 2:** Macronutrient, energy, and phenylalanine content of four commercial protein supplements and the three snacks (ink A, B, and C), based on product labels for the commercial substitutes and ingredient labels for the snacks developed in this work.

Content	PKU GOLIKE - tropical bar	Glytactin COMPLETE ® 15 Fruit Frenzy	XPhe Hello	GMP UP Afenil bars - coconut	Food inks developed		
					A	B	C
Protein content (g/100 g)	17.0	19.0	25.0	16.7	24.1	27.7	31.3
Fat (g/100 g)	0.0	12.0	0.4	7.2	34.5	34.7	34.9
Carbohydrate (g/100 g)	52.0	38.0	39.0	50.0	30.9	25.6	20.3
Sugar (g/100 g)	34.0	25.0	30.0	20.0	10.0	7.8	5.6
Fibers (g/100 g)	1.6	1.0	13.0	8.4	0.37	0.42	0.47
Energy (kcal/100 g)	280.0	405.0	290.0	349.0	485.7	496.0	506.4
Phe content (mg/100 g)	25.0	40.0	36.0	21.0	43.2	49.2	55.2

Medical protein substitutes are specifically designed to supply all essential AAs except Phe, often contain Tyrosine, which is needed in PKU, and are generally fortified with vitamins and minerals (since PKU diets lack many). In general, the most important aspects to consider in a protein supplement are the Phe content (should be as low as possible) and the protein content (as high as possible and with adequate formula in terms of AAs) but the energy content also plays a role in low-energy (for those needing to manage weight) or high-energy (for those needing growth catch-up or extra calories) diets. Selecting the right formula depends on several factors (age, growth patterns, activity level, weight goals) but in general, lowering sugar and fat contents and increasing the fiber content contribute to a better nutritional composition (Tosti et al., 2024).

### 3.2 Thermal properties

**Fig. 1:**
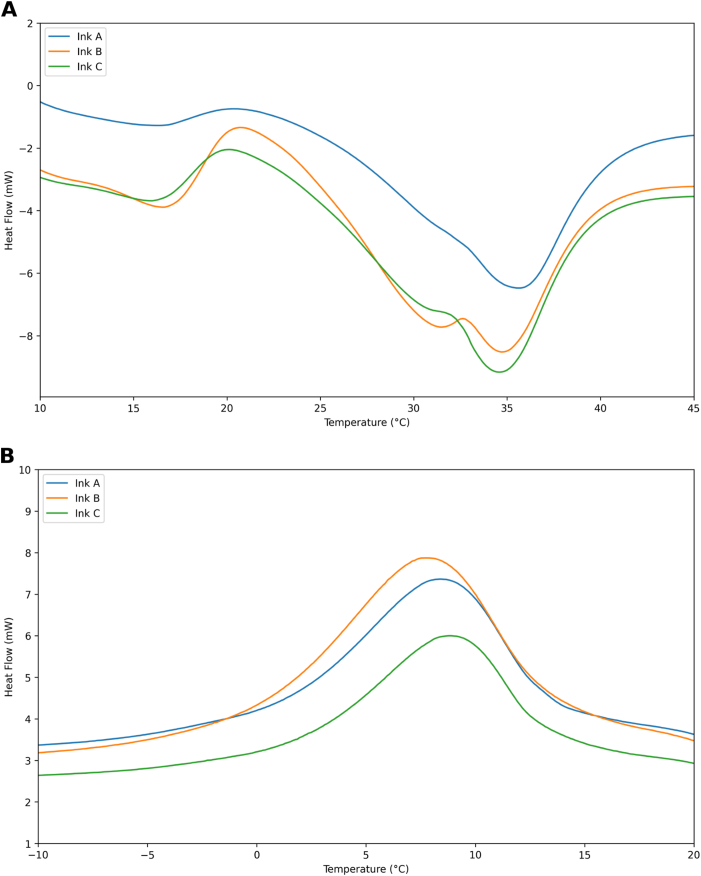
Melting (A) and crystallization (B) profiles of the formulated inks.

Thermal analysis was carried out to characterize the melting behaviour of the lipid phase in the GMP-based inks, since the onset and peak melting temperatures define the temperature window suitable for extrusion-based 3D printing, while the melting enthalpy reflects the extent of fat crystallinity, which is expected to influence both the mechanical stability of the printed objects and their melt-in-mouth sensation. The thermal behavior of the formulated inks was analyzed using DSC melting and crystallization curves (Fig. 1). The corresponding thermal parameters are summarized in Table 3.

**Table 3:** Thermal parameters of formulated inks. Different letters indicate statistically significant differences among formulations.

	Tonset (°C)	Tendset (°C)	Tpk (°C)	ΔH (J/g)
Melting				
A	20.79^a^ ±0.35	45.59^a^ ±0.53	35.52^a^ ±0.42	28.18^a^ ± 3.86
B	20.86^a^ ±0.40	43.57^a^ ±0.85	34.03^a^ ±1.26	21.91^b^ ± 2.07
C	20.26^a^ ±0.27	43.49^a^ ±0.64	34.45^a^ ±0.24	22.23^b^ ± 3.18
Crystallization				
A	20.70^a^ ±0.36	−5.88^a^ ±0.24	8.43^a^ ±0.07	21.04^a^ ±0.42
B	21.41^a^ ±0.30	−6.44^a^ ±0.42	8.18^a^ ±0.43	21.56^a^ ±2.47
C	21.25^a^ ±0.01	−5.34^a^ ±0.06	8.46^a^ ±0.39	21.67^a^ ±0.32

The melting curves (Fig. 1A) exhibited characteristic phase transitions across all formulations, with T_onset_ values ranging from 20.26 °C to 20.86 °C. This indicated relatively consistent initiation of melting across the inks. However, differences in the T_endset_ and T_pk_ values suggest variations in the final stage of melting. Formulation A displayed the highest peak temperature (T_pk_ = 35.52 °C) and significantly different enthalpy (ΔH = 28.18 J/g) compared to the other two formulations, suggesting a more ordered crystalline structure. The observed reduction in ΔH when the XPhe enjoy^20^ GMP concentration exceeds 46 % suggested a significant impact of protein inclusion on the thermal behavior of the inks. The crystallization curves (Fig. 1B) demonstrated well-defined exothermic peaks across all formulations, indicating similar crystallization processes. The T_onset_ values ranged from 20.7 °C (formulation A) to 21.41 °C (formulation B), showing no significant differences in crystallization initiation. The enthalpy of crystallization (ΔH) remained relatively stable across formulations (21.04–21.67 J/g). These values showed no statistically significant differences, suggesting that variations in GMP content did not substantially alter the total energy released during the crystallization.

### 3.3 Rheological characteristics

**Fig. 2:**
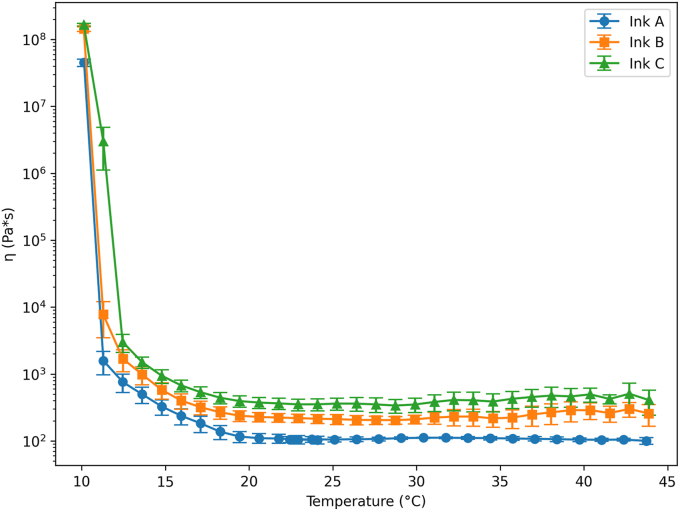
Viscosity curves of inks (Formulations A, B, and C) as a function of temperature.

Rheological analyses were conducted to determine the temperature- and shear-dependent viscosity behaviour of the GMP-based inks, as these properties are directly linked to extrusion feasibility, filament stability during deposition, and the ability of the printed structure to retain its shape. Fig. 2 indicates the viscosity's curves for the three formulated inks.

Viscosity measurements showed that the viscosity of all three inks significantly decreased as temperature increased. This trend is particularly evident below 15 °C, so that ink C, which had the highest concentration of XPhe enjoy^20^ GMP (52 %), exhibited the highest viscosity, followed by ink B (46 % XPhe enjoy^20^ GMP) and ink A (40 % XPhe enjoy^20^ GMP). The higher viscosity observed in inks with increased XPhe enjoy^20^ GMP content suggests that XPhe enjoy^20^ GMP have a significant impact into structuring the ink. Notably, beyond 20 °C, the viscosity curves for all inks stabilized. Ink C consistently had a higher viscosity than inks A and B, indicating the effect of GMP at high concentrations, which leads to network stabilization even under increased thermal conditions.

### 3.4 Printing accuracy and colorimetric parameters

**Fig. 3:**
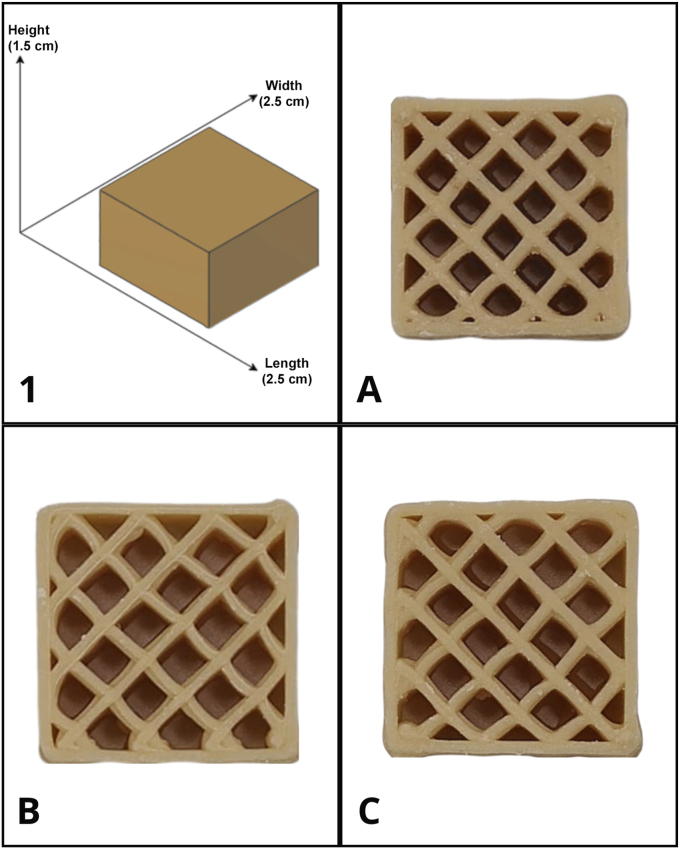
(1) 3D model of printed object with dimensions (Length: 2.5 cm; Width: 2.5 cm; Height: 1.5 cm). (A) Printed object using Ink A, (B) Printed object using Ink B, and (C) Printed object using ink C.

The printing accuracy of the formulated inks was measured to assess the structural fidelity and the reproducibility of 3D printed objects. Examples of the printed objects are reported in Fig. 3.

As shown in Table 4, all printed objects achieved satisfactory dimensional fidelity. The actual printed dimensions closely matched the designed specifications of 2.5 × 2.5 × 1.5 cm. The infill pattern analysis demonstrated that the achieved infill percentage was consistent with the predefined setting of 40 % across all formulations.

**Table 4:** Area and infill accuracy of printed objects from the A, B and C inks obtained by image analysis.

Element (%)	Ink A	Ink B	Ink C
**Area accuracy**	95.83 ± 1.77	95.63 ± 0.66	96.72 ± 0.58
**Infill accuracy**	90.32 ± 3.97	92.75 ± 2.63	92.20 ± 2.51

No significant differences were observed among the printed objects for both area and infill accuracy. Ink C exhibited the highest area accuracy (96.72 ± 0.58 %), followed by ink A (95.83 ± 1.77 %) and B (95.63 ± 0.66 %).

**Fig. 4:**
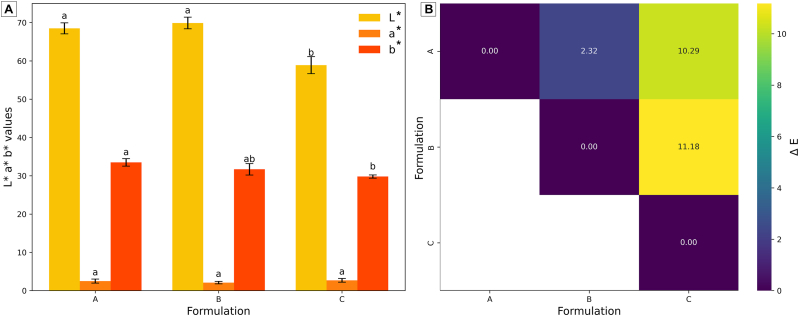
L∗a∗b∗ color space coordinates (A) and heatmap (B) of ΔE for the three printed objects. Different letters represent a significant difference between formulations.

The colorimetric analysis of the three printed objects (A, B, and C) revealed significant differences in the color parameters L**∗**, a**∗**, and b**∗**, as shown in Fig. 4. Overall, the three printed objects showed a fairly light color (L∗ in the range 57–72) with a strong yellow (b∗ in the range 29–35). The L**∗** values (lightness) were highest for printed objects A and B, with no statistically significant differences between them (both marked as "a"), while printed object C exhibited a significantly lower L**∗** value, indicating a darker appearance. Similarly, the a**∗** values (green-red spectrum) showed slight variations: printed object A had the highest a**∗** value, differing significantly from printed object C, while printed object B displayed intermediate values. The b**∗** parameter (blue-yellow spectrum) followed a similar trend, with printed objects A and B showing higher b**∗** values compared to printed object C, suggesting a more yellowish tone. The ΔE values, representing the total color differences between printed objects, are visualized in the heatmap (right panel). The greatest color difference (ΔE = 11.18) was observed among printed objects B and C, followed by the difference among A and C (ΔE = 10.29). In contrast, printed objects A and B showed minimal color difference (ΔE = 2.32). These results indicate that printed object C differed significantly from printed objects A and B in terms of color attributes. The ΔE values further confirmed this trend, showing that printed object C is visually distinct from the other printed objects.

### 3.5 Wetting behavior

**Fig. 5:**
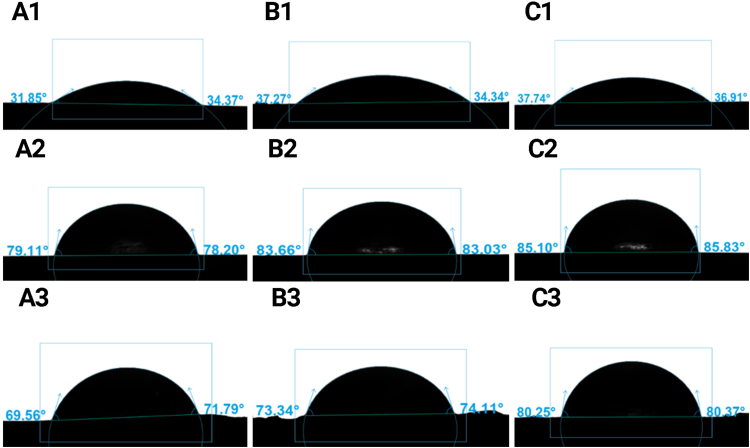
Sessile drop pictures of the water drop taken after 60 s from deposition for the three formulations (A, B and C) studied for non-tempered (1), tempered (2) inks, and for 3D printed objects (3).

The wettability of the 3D printed objects and tempered, and non-tempered inks was studied by contact angle measurements using the sessile drop method with water. Fig. 5 shows the water droplets after 60 s from deposition. Regardless of the formulation, the wettability of the printed objects was similar to that of the tempered ink and was higher compared to the non-tempered ink.

**Fig. 6:**
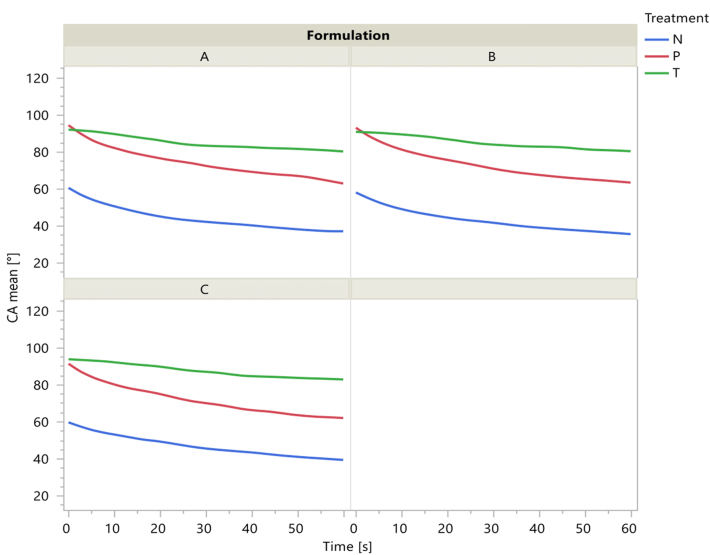
Contact angle measurements of non-tempered (N) and tempered (T) inks 3D printed (P) objects, for A, B and C formulations. The curves were obtained as spline interpolations of the mean contact angle (arithmetic mean of left and right contact angle) of replicated measurements. Multiple comparisons of the means at 60 s showed statistically significant differences between the treatments.

Fig. 6illustrates the data of contact angle measured from sessile drop pictures collected as a function of deposition time. The data show similar time trends for the three samples with differences in the wetting behavior induced by the sample treatment. Tempered inks showed the highest values of contact angle, decreasing from approximately 94 to 80° as a function of time, thus with a decrease of 14°. Non-tempered inks presented a much higher wettability, with contact angles decreasing from about 61 to 35° as a function of time with a variation of 26°. These results align with those of other researchers Reinke et al. (2015) who have studied the wettability of chocolate by water. They observed that non-tempered chocolate exhibits higher wettability and a greater decrease in contact angle compared to tempered chocolate. The wettability results showed contact angles of 3D-printed objects were more similar to the results of tempered inks. They presented slightly lower contact angles than the tempered ones, but they were still much higher than the non-tempered inks data. This could confirm that the 3D printing process (at 32 °C) only slightly altered the crystallization state of the fat because the stable crystals (V form) formed during the tempering process start melting at temperatures in the 30–32 °C range (Reinke et al., 2015).

### 3.6 Mechanical properties

The mechanical properties of the printed objects were evaluated through breaking stress and Young's modulus measurements, as shown in Table 5.

**Table 5:** Young modulus and breaking stress of the 3D-printed objects obtained from the formulated inks. Data did not show significant differences statistically.

3D-printed objects	Young Modulus (MPa)	Breaking stress (Pa)
A	5.59 ± 1.03	537.2 ± 82.01
B	6.79 ± 0.78	615.04 ± 70.52
C	6.54 ± 2.23	563.89 ± 36.3

Young's modulus, which reflects the stiffness of the material, ranged from 5.59 ± 1.03 MPa for 3D-printed object A to 6.79 ± 0.78 MPa for 3D-printed object B, with 3D-printed object C showing an intermediate value of 6.54 ± 2.23 MPa. Breaking stress values varied between 537.2 ± 82.01 Pa (A), 615.04 ± 70.52 Pa (B), and 563.89 ± 36.3 Pa (C). No statistically significant differences were observed among 3D-printed objects for either parameter. These results have direct implications for 3D printing performance, where balancing mechanical strength and flexibility is crucial in order to achieve stable, yet extrudable structures suitable for personalized food applications.

### 3.7 Sensory analysis

**Fig. 7:**
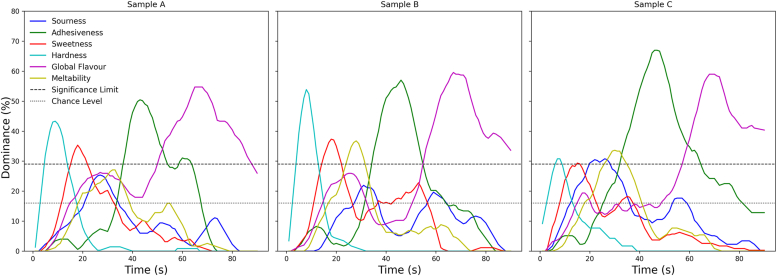
Temporal Dominance of Sensations (TDS) curves for the 3D printed objects.

Fig. 7 indicates the results of the TDS analysis for each of the 3D printed objects with different formulations.

The incorporation of GMP in the inks played a crucial role in modulating sensory perception. Each 3D printed object exhibited distinct dominance patterns for texture, flavor, and mouthfeel attributes, and these differences were analyzed further using McNemar's test for paired comparisons every 10 s (Supplementary material T1). In general, overall flavor and adhesiveness emerged as the most dominant attributes across all 3D printed objects, exceeding the significance threshold for extended periods. 3D printed objects with higher GMP content exhibited longer-lasting flavor and adhesiveness in the TDS curves. In fact, statistical analysis confirmed that adhesiveness was significantly different between 3D printed objects, particularly between A vs B (p < 0.001, 31–40 s; p = 0.001, 41–50 s) and A vs C (p < 0.001 from 21 to 50 s), suggesting that higher GMP content (B: 46 %, C: 52 %) increased oral adhesiveness. This is consistent with our previous rheological analysis, where higher GMP concentrations contributed to stronger structuring. Sweetness perception varied significantly, especially in the early stages of mastication (11–20s and 21–30 s). 3D printed object A, which contained the highest tagatose content (16.2 %), showed significantly higher sweetness perception compared to B and C (p < 0.001, 41–60 s for A vs B; p < 0.001, 21–30 s and 31–40 s for A vs C). The early higher dominance of hardness in printed object C could be related to its higher GMP content, which may have led to increased structural resistance upon initial mastication. However, as the object broke down, hardness perception declined rapidly in all 3D printed objects, aligning with the mechanical tests, where breaking stress did not show major differences between 3D printed objects. Meltability showed notable differences in the middle phase of oral processing (21–70 s). McNemar's test confirmed significant differences between A vs B (p < 0.001, 61–70s) and A vs C (p < 0.001, 51–60s). These findings show that 3D printed objects with lower tagatose and higher GMP (C > B > A) exhibited greater structural integrity over time, delaying the perception of meltability. Finally, acidity emerged as a significantly dominant sensation only in printed object C, particularly in the early to mid-stages of oral processing. While sourness did not exceed the dominance threshold in printed objects A and B, it was significantly more perceivable in C, as confirmed by McNemar's test for A vs C (p < 0.001, 11–20s) and B vs C (p < 0.001, 11–30s).

## 4 Discussion

Medical protein substitutes are specifically designed to supply all essential AAs except Phe, often contain Tyrosine, which is needed in PKU, and are generally fortified with vitamins and minerals (since PKU diets lack many) (Ilgaz et al., 2021). The most important aspects to consider in a protein supplement are the Phe content (should be as low as possible) and the protein content (as high as possible and with adequate formula in terms of AAs). We used a commercial GMP-based formula as a base for ink development. This ensured the supply of a balanced AAs formula with a content of Phe as low as 0.1 g/100 g of formula (such Phe level is likely due to the presence of GMP in the product, which is difficult to produce completely free of Phe). To obtain a shelf-stable and printable material, a confectionery ink analogous to chocolate was formulated by adding cocoa butter as the lipid base, a sweetener to provide sweetness, a flavoring agent to enhance sensory attributes, and lecithin as an emulsifier to improve ingredient dispersion. Tagatose was used as the sweetener instead of sucrose in order to reduce the caloric and glycemic impact of the formulation, while maintaining sweetness and technological functionality comparable to sucrose. Ingredient selection was guided by assessing phenylalanine content alongside nutritional quality to ensure a balanced final product. Cocoa butter, was used to make the product extrudable. It should be noted that cocoa butter contains about 60 % of saturated fatty acids, and a high intake of saturated fat is generally not recommended in current dietary guidelines. However, the 3D-printed products are conceived as specialized medical foods for PKU, to be consumed in controlled portions within a low-phenylalanine diet and under medical/nutritional supervision, rather than as freely consumed snack. Tagatose, another Phe-free product, served as a low-glycemic sweetener with prebiotic benefits. For this reason, it was used instead of sucrose to maintain sweetness and technological functionality. Pineapple juice powder added with maltodextrins, a low-Phe fruit mix powder, was chosen as a flavoring agent. The Phe content of the snacks varied in the range 40–52 mg Phe/100 g of which only 3.2 mg/100 g came from the pineapple powder, while the rest derived from the formula.

The thermal analysis revealed significant insights into the complex interactions between GMP and the lipid matrix. The observed reduction in melting enthalpy when XPhe enjoy^20^ GMP concentration exceeded 46 % suggested a fundamental alteration in the crystalline structure of the cocoa butter matrix. Studies on protein-lipid systems indicate that proteins can modulate nucleation and crystal growth, leading to a less ordered and more heterogeneous crystalline network. In this case, the GMP contained in XPhe enjoy^20^GMP,may be disrupting cocoa butter's crystalline packing, resulting in a broader melting transition and reduced enthalpy (Aruchunan et al., 2025). The phase separation hypothesis proposed in previous studies may also explain the behavior observed in our study, as GMP may create heterogeneous domains within the lipid phase of the ink, preventing the formation of more stable crystalline forms. Additionally, dairy proteins have been shown to influence molecular mobility which could potentially alter the crystallization kinetics of the matrix (Gloria Hernández et al., 2011).

The increased viscosity observed with higher GMP concentrations can be attributed to the protein's ability to form intermolecular networks through hydrogen bonding and electrostatic interactions. Unlike whey proteins, GMP does not form covalent disulfide bonds but still contributes significantly to matrix structuring through non-covalent interactions, as demonstrated by Godoy-García et al. (2020). Soleimani et al. (2024) stated that increasing the protein content by adding GMP concentrations enhanced viscosity of cream cheese. Across 35-21 °C all inks showed a broad viscosity plateau (≈110, 227, 365 Pa s), followed by a steep rise on further cooling (to ≈760, 1688, 3100 Pa s at around 12 °C), consistent with cocoa-butter crystallization. Compared with 3D-printed chocolate systems around 33 % cocoa butter, our apparent viscosities are markedly higher, whereas Mantihal et al. (2017) reported lower values under comparable fat levels and higher shear.

The melting and rheological properties of the three formulations help to explain their successful 3D printability. The DSC melting profiles, with onset and peak temperatures close to the printing temperature, indicate that the lipid phase is partially molten during extrusion but rapidly crystallizes upon cooling to room temperature. This behaviour creates a favorable compromise between flowability through the nozzle and post-deposition solidification, limiting spreading and collapse of the printed filaments. In parallel, the viscosity-temperature behaviour shows that at the printing temperature the inks present sufficiently low viscosity to allow continuous extrusion, whereas the sharp increase in viscosity on cooling supports layer stacking and self-support of the structure.

Although the formulation interval investigated did not lead to significant differences in several measured parameters, a clear increase in viscosity was observed with increasing GMP content. This rheological change had a direct impact on both chocolate processing and 3D printability. Higher GMP levels made chocolate refining and molding more difficult, increasing the risk of air entrapment during the preparation of chocolate cylinders. During printing, the formulation with the highest GMP content required longer pre-heating times and frequently resulted in incomplete prints due to inconsistent extrusion and air bubbles. These processing constraints indicate that further increases in GMP content beyond the tested range would likely exceed the practical limits of chocolate handling and extrusion-based 3D printing, rather than improving functional performance.

Regarding the mechanical properties of the 3D printed objects, the GMP concentration had no significant impact on Young moduli or breaking stress. Despite the absence of significant differences across GMP levels, our prints are in line with chocolate systems reported in the 3D-printing literature. In printed chocolate, internal architecture is a primary lever, since higher infill percentages and stiffer infill topologies (like honeycomb shapes) raise hardness/strength relative to low-infill or more porous designs. Taken together, the lack of GMP-level effects in our study suggests that, within the tested formulation window, bulk stiffness is far more sensitive to designed porosity/infill than to protein concentration, mirroring trends seen for aerated/architected chocolates (Bikos et al., 2021, 2023; Mantihal et al., 2019).

The successful 3D printing of all three inks demonstrated the technological feasibility of producing personalized GMP-based protein supplements using extrusion-based additive manufacturing. Printing accuracy results, showing dimensional fidelity close to the designed specifications, align with recent advances in food 3D printing technology (Jiang et al., 2019). The selection of GMP as the primary protein source is consistent with extensive research demonstrating its superior acceptability and physiological benefits compared to traditional amino acid mixtures (Russo et al., 2025). Van Calcar and Ney (Van Calcar and Ney, 2012) established that GMP-based formulations have significant advantages in terms of taste, texture, and patient compliance. These factors are critical for long-term dietary adherence in the management of PKU. The wetting behaviour of the 3D printed objects was similar to that of the tempered inks, suggesting that the thermal history of the material during the 3D printing process did not substantially alter the characteristics of the tempered ink. During 3D printing, as reported by researchers (Va et al., 2023), the polymorphic crystal state of cocoa butter in chocolate can change, resulting in poor quality products. However, this is provided that the ink has undergone a weak tempering process before printing. Proper tempering of chocolate and cocoa butter-based products is essential to obtain a final product with a smooth and glossy finish, solid at room temperature, and with the correct sensory attributes. Non-tempered or improperly tempered chocolate can lead to fat blooming, that is, the migration of fat to the surface of chocolate resulting in superficial color change and uneven pattern formation (Briones and Aguilera, 2005), which negatively affects consumer perception. The lack of visible bloom on the 3D printed objects during storage at room temperature provided further evidence that the tempering process was properly conducted and that the printing parameters maintained the integrity of the object's structure, producing shelf stable 3D printed objects.

The sensory evaluation conducted through TDS methodology provided key insights into how formula variables, particularly GMP and tagatose content, shape mouthfeel, flavor dynamics, and overall acceptability of 3D-printed protein substitutes for PKU patients. Consistent with previous findings on GMP's influence on appetite response and protein palatability (Warner et al., 2024) our results confirmed its important role in modulating sensory perception. 3D printed objects with higher GMP content (B and C) showed prolonged adhesiveness dominance, reflecting a more cohesive and persistent oral texture. This effect is likely due to GMP's hydrocolloid-like behavior, as shown in previous research (Godoy-García et al., 2020; Dickinson, 2001), where GMP contributes to matrix structuring through electrostatic and hydrophilic interactions (Warner et al., 2024). This trait is beneficial for patients with PKU, as it can improve palatability, reducing the aversion commonly reported with synthetic amino acid supplements (Proserpio et al., 2019).

Sweetness dominance was most evident in 3D printed object A, which contained the highest tagatose concentration. Tagatose, a low-calorie natural sweetener, is known for its clean, sucrose-like taste and its role in masking bitterness and metallic notes often associated with free amino acids. Its effect diminished quickly in the TDS curves, highlighting the known rapid solubilization and clearance of tagatose in the oral cavity, confirming its similarity with sucrose in imparting sweetness to the food.

Interestingly, meltability was also influenced by the protein-sweetener ratio. 3D printed object C, the richest in GMP but lowest in tagatose, exhibited great structural resistance during the initial mastication phase, which was later followed by a delayed meltability profile. These findings align with Wilbanks et al. (2022), who showed that higher casein content in milk gels leads to increased mechanical strength and slower oral disintegration.

Sourness perception, though less dominant overall, became statistically significant only in printed object C during early mastication stages. The use of pineapple juice powder as a flavoring agent, combined with lower sweetness masking in C, likely led to the dominance of acidic notes. This taste imbalance may negatively affect consumer perception (Delarue and Sieffermann, 2004), particularly in pediatric or sensitive populations, and highlights the need for taste-balancing strategies (e.g., sweet-acid pairings) to optimize palatability. Using sour-suppressing aromas (Spence, 2022) could help mitigate this challenge.

Extended global flavour perception in printed objects B and C further underscores GMP's dual role as both a nutritional protein and a flavored carrier, capable of mimicking the sensory complexity of conventional protein foods. These results align with prior studies that emphasize GMP's mild and pleasant taste and its superior sensory profile over synthetic amino acid blends (Van Calcar and Ney, 2012; Proserpio et al., 2018).

On the one hand, our findings reinforced the importance of targeted sensory design in medical nutrition, especially in populations with elevated risk of dietary non-compliance. On the other hand, the sensory results underlined the added value of 3D printing technology, which not only allows for precise control over nutritional content but also offers an additional layer of customization, enabling texture manipulation, flavor release, and visual appeal through geometric design (Goyanes et al., 2019; Carou-Senra et al., 2024).

Beyond sensory benefits, GMP may confer physiological advantages over free amino acid mixtures. Recent research by Warner et al. (2024) reported that GMP intake influences appetite regulation and gut hormone responses, potentially contributing to better satiety and metabolic outcomes. This is particularly relevant for PKU patients, who often struggle with hunger and dietary monotony due to the restrictive nature of their prescribed diets. Furthermore, emerging evidence suggests that GMP may modulate oxidative stress and inflammation, offering broader health benefits within PKU dietary management.

## 5 Conclusion

This study suggests that 3D printing may be a feasible approach for producing customized, palatable medical foods for PKU patients, with GMP serving as a key functional protein. Our cocoa butter-based inks were not only suitable for extrusion-based 3D printing, but also nutritionally and sensorially appropriate for PKU patients. By modulating GMP and tagatose levels, it is possible to fine-tune mechanical strength, thermal stability, and sensory appeal of the printed snacks to meet individual dietary and compliance needs. The high printing accuracy and favorable sensory profiles underline the potential of such products for daily use, supporting the shift toward personalized medical foods. Future studies should explore patient trials for acceptability evaluation and potential integration into hospital or home-based digital nutrition systems. Finally, this innovative approach may enhance treatment adherence and overall quality of life for individuals living with PKU.

## Ethical approval

Ethical approval was granted by the ethics committee (CERSUB) of University of Naples Federico II (PG/2024/0137,296 October 29, 2024). The study was conducted in agreement with the guidelines of the Declaration of Helsinki, the rules of “Personal Data Protection Code”, Reg. EU 2016/679 of the European Parliament and the Italian ethical requirements on research activities and personal data protection (D.L. 30.6.03 n. 196). Informed consent was obtained from the eight trained assessors involved in the sensory evaluation, ensuring respect for privacy, participants’ rights and any information related to allergy or intolerances of participants to the ingredients of our formulations.

## Credit author statement

**Marcello Fidaleo:** Conceptualization, Writing original draft, Methodology, Resources, Supervision, Funding acquisition**. Zohreh Baratian Ghorghi:** Data curation, Formal analysis, Software, Validation, Writing – review and editing. **Russo Giovanni Luca:** Data curation, Formal analysis, Software, Validation, Writing – review and editing. **Annachiara Ferraioli**: Data curation, Formal analysis, Software. **Silvana Cavella**: Conceptualization, Visualization, Investigation, Writing- Reviewing and Editing, Validation, Supervision, Resources. **Rossella Di Monaco:** Conceptualization, Visualization, Writing- Reviewing and Editing, Supervision, Project administration, Funding acquisition.

## Funding

This study was funded by the 10.13039/501100000780European Union – Next Generation EU - Italian Ministry of University and Research (MUR), project 202274NMW3 (PRIN-2022), entitled “development of Medical foods with improved sensory properties and personalized nutritional content for patients with phenylketonuria through extrusion-based 3D prInTING” (M3DITING).

The authors are thankful to: Carlo Vanni, Head of R&D, BU Ice Cream from Casa Optima S.p.A. (San Clemente, RN, Italy) for the generous donation of cocoa butter, pineapple powder, and lecithin, and for his valuable insights and helpful suggestions, which significantly enriched the quality of this work; Dr. Carlo Dionisi-Vici from the Bambino Gesù Children's Research Hospital (Rome, Italy) for his valuable insights and helpful suggestions; Dr. Vincenzo Merolla from Mamoxi S.r.l. (Torino, Italy) for his valuable insights and helpful suggestions; Bonumose Inc. (Charlottesville, 10.13039/100000738VA, USA) for the generous donation of tagatose; and Luisa Liguori for her support during the experiments.

## Declaration of competing interest

The authors declare that they have no known competing financial interests or personal relationships that could have appeared to influence the work reported in this paper.
