# Supplementary material for: Personalized, digitally designed 3D-Printed protein substitutes: Advancing medical food for PKU patients
Source: Curr Res Food Sci. 2026 Jan 19;12:101314. doi: 10.1016/j.crfs.2026.101314 (PMC12860630; doi:10.1016/j.crfs.2026.101314)
Supplement: Multimedia component 1 [file mmc1.docx]

**Supplementary material**

**Table S1**. Results of Mcnemar test conducted every 10 seconds on the dominance table of the temporal dominance of sensations conducted.

| **A vs B** | | | | | | |
| --- | --- | --- | --- | --- | --- | --- |
| **Time frame** | **Acidity** | **Adhesiveness** | **Sweetness** | **Hardness** | **Global flavour** | **Meltability** |
| **1-10s** | >0.8 | >0.8 | >0.8 | >0.8 | >0.8 | >0.8 |
| **11-20s** | <0.001 | 0.004 | 0.403 | 0.012 | >0.8 | 0.281 |
| **21-30s** | 0.002 | 0.011 | 0.305 | 0.549 | 0.771 | <0.001 |
| **31-40s** | 0.145 | 0.001 | 0.741 | >0.8 | <0.001 | 0.022 |
| **41-50s** | 0.766 | 0.103 | <0.001 | >0.8 | <0.001 | 0.027 |
| **51-60s** | 0.012 | 0.461 | <0.001 | >0.8 | 0.001 | <0.001 |
| **61-70s** | <0.001 | <0.001 | 0.006 | >0.8 | 0.731 | <0.001 |
| **71-80s** | 0.512 | >0.8 | >0.8 | >0.8 | 0.072 | >0.8 |
| **81-90s** | >0.8 | >0.8 | >0.8 | >0.8 | 0.202 | >0.8 |
| **A vs C** | | | | | | |
| **1-10s** | >0.8 | >0.8 | >0.8 | 0.743 | >0.8 | >0.8 |
| **11-20s** | <0.001 | 0.625 | 0.691 | <0.001 | >0.8 | 0.608 |
| **21-30s** | 0.074 | <0.001 | <0.001 | <0.001 | <0.001 | 0.049 |
| **31-40s** | 0.206 | <0.001 | 0.021 | >0.8 | <0.001 | 0.116 |
| **41-50s** | 0.141 | <0.001 | 0.029 | >0.8 | 0.044 | 0.358 |
| **51-60s** | 0.001 | <0.001 | 0.188 | 0.728 | <0.001 | <0.001 |
| **61-70s** | <0.001 | 0.348 | >0.8 | >0.8 | 0.526 | 0.029 |
| **71-80s** | <0.001 | >0.8 | >0.8 | >0.8 | 0.011 | >0.8 |
| **81-90s** | >0.8 | >0.8 | >0.8 | >0.8 | 0.026 | >0.8 |
| **B vs C** | | | | | | |
| **1-10s** | >0.8 | >0.8 | >0.8 | 0.483 | 0.724 | >0.8 |
| **11-20s** | <0.001 | 0.027 | 0.721 | 0.005 | >0.8 | 0.533 |
| **21-30s** | <0.001 | <0.001 | 0.005 | 0.005 | 0.128 | 0.159 |
| **31-40s** | 0.719 | 0.017 | 0.044 | >0.8 | 0.497 | <0.001 |
| **41-50s** | 0.063 | 0.003 | <0.001 | 0.613 | 0.001 | 0.222 |
| **51-60s** | 0.193 | <0.001 | <0.001 | >0.8 | 0.101 | >0.8 |
| **61-70s** | 0.002 | <0.001 | 0.003 | >0.8 | 0.235 | 0.104 |
| **71-80s** | <0.001 | <0.001 | 0.070 | >0.8 | 0.738 | >0.8 |
| **81-90s** | >0.8 | >0.8 | 0.625 | >0.8 | 0.259 | >0.8 |
